# Supplementary material for: One-step fabrication of crystalline metal nanostructures by direct nanoimprinting below melting temperatures
Source: Nat Commun. 2017 Mar 28;8:14910. doi: 10.1038/ncomms14910 (PMC5379067; doi:10.1038/ncomms14910)
Supplement: Supplementary Information — Supplementary Figures, Supplementary Tables and Supplementary Reference. [file ncomms14910-s1.pdf]

Supplementary Tables

Supplementary Table 1| Measured diameter of as-thermoplastically formed Au discs and calculated mean forming pressure.

Sample No.	1	2	3	4	5
Holding time (s)	60	240	480	1200	3600
Final diameter (mm)	5.00	5.21	5.22	5.28	5.33
Mean forming pressure (MPa)	509	476	467	457	448

Supplementary Table 2| Observed wavenumbers (cm^{-1}) of SERS in crystal violet (CV) and their assignment

SERS (cm^{-1})	Vibrational assignment [1]
420, 442	Out-of-plane ring skeletal vibration + out-of-plane C^+-phenyl
524, 558, 608	Ring skeletal vibration of radial orientation
660, 722, 765, 802	Out-of-plane ring (C-H) bend
913	Ring skeletal vibration of radial orientation
979, 1174	In-plane ring C-H bend
1294	Ring C-C stretching
1365, 1384	N-phenyl stretching
1448, 1478	Ring C-C stretching + ring deformation
1537, 1585, 1620	Ring C-C stretching

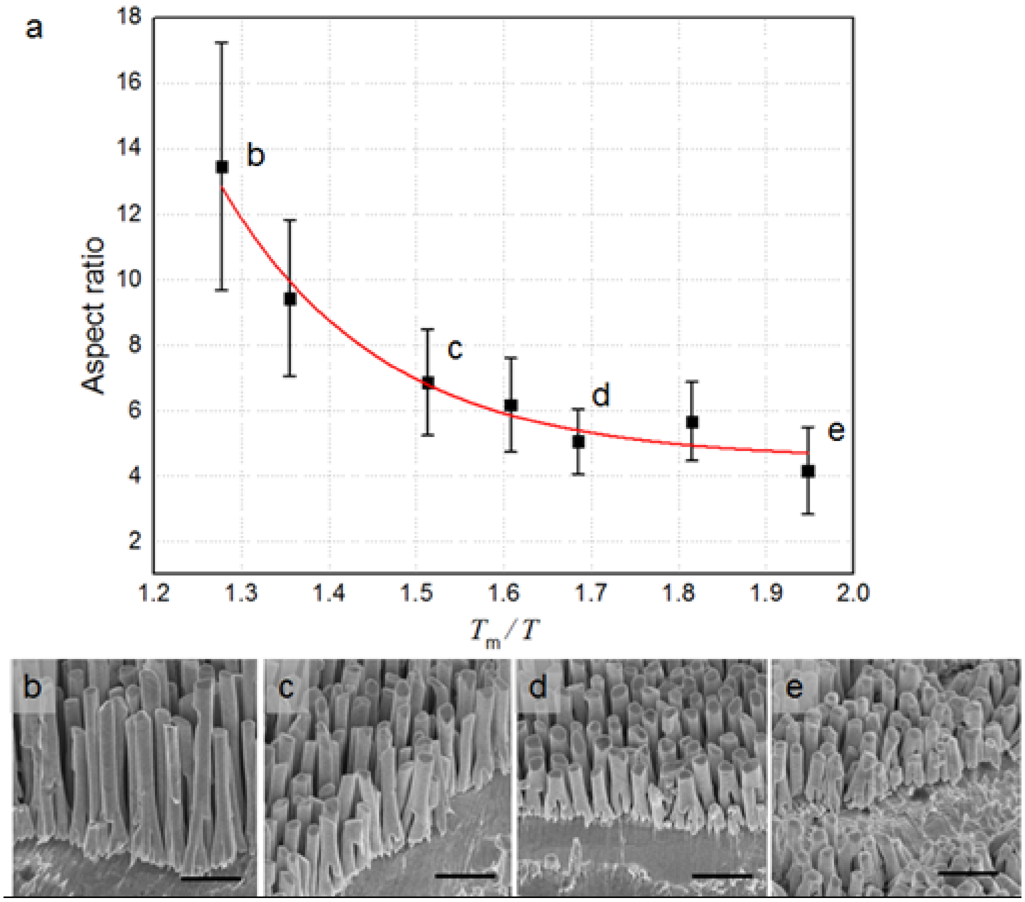

Supplementary Figure 1| Processing temperature dependent length of imprinted Au nanorods. a, Aspect ratios of Au nanorods versus processing temperatures, where seven Au short rods (17.0 ± 0.2 mg) were prepressed at 520℃ to get flat discs with thickness of ~ 0.65 mm. The Au flat discs were subsequently superplastically formed into 100 nm Al_2O_3 templates at 413, 463, 520, 558, 610, 713, 773℃, respectively, where all the samples were loaded to 1.5 kN and then held for 100s. The length of Au nanorods at the center of each sample are measured under SEM by tilting the sample stage for 50 degrees (**b-e,** scale bar, 1 μm). Further fitting the experimental data with eq. 2 gives $L/d = 4.50 + 9150.80\exp(-5.48T_m/T)$ (red line in **a**). The error bars are defined as s.d. and obtained by measuring at least 10 nanowires for each sample.

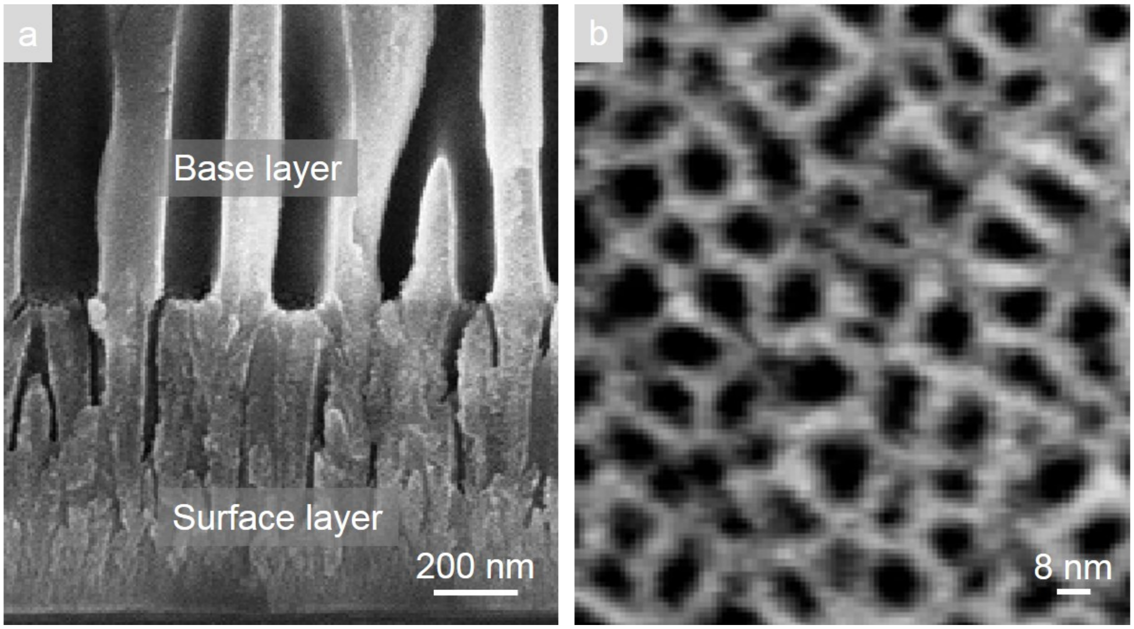

Supplementary Figure 2| SEM micrographs of hierarchical Al_2O_3 template with branched nanopores in its surface layer (from Synkerainc). a, Hierarchical structures from the view of section. **b**, Smallest nanopores in the outmost surface of the Al_2O_3 template is ~ 8 nm.

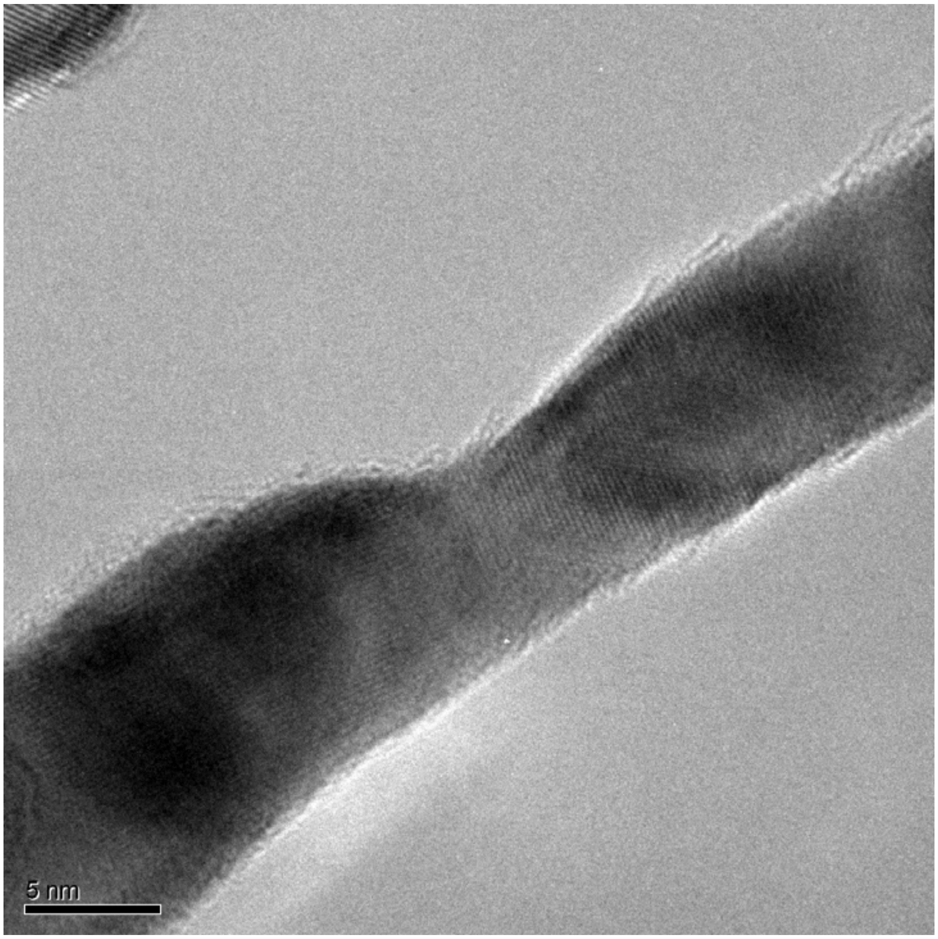

Supplementary Figure 3| The smallest Au nanorod fabricated by SPNI. High-resolution TEM imaging of an Au hierarchical nanostructure at its smallest part shows a replicated size of ~ 8 nm, which is in consistent with the used hierarchical Al_2O_3 template (Fig. 3).

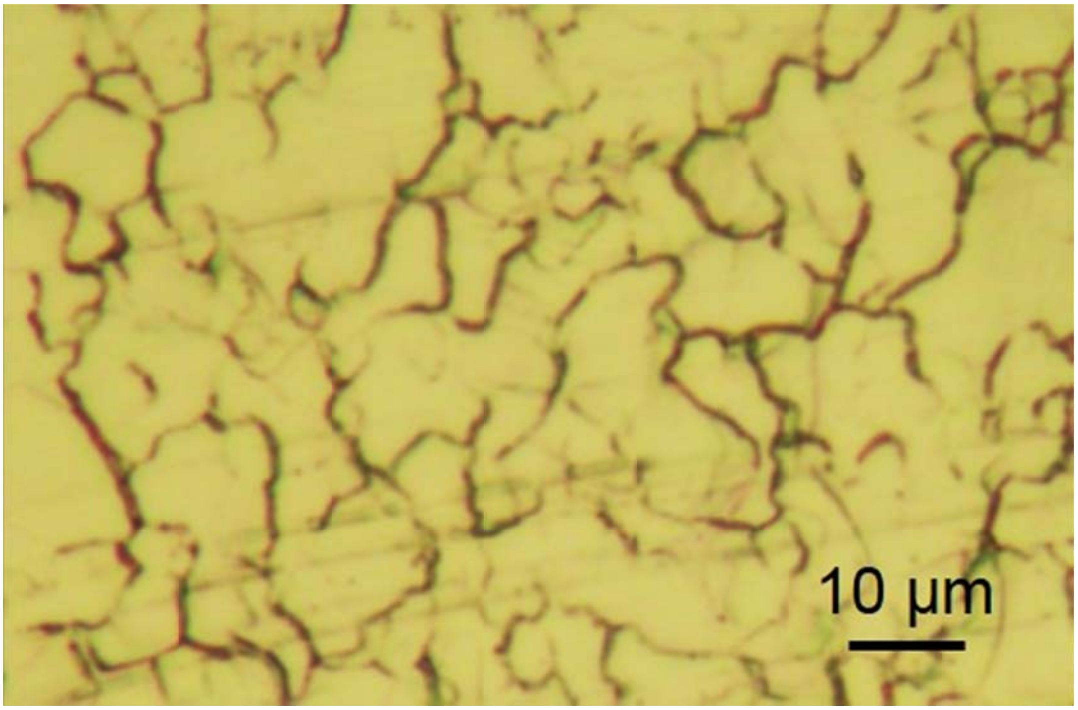

Supplementary Figure 4| Grain size of bulk Au. A piece of bulk Au was superplastically formed without mold at ~ 500℃, under an applied force of 5 kN and holding for 4 min. The optical micrograph of the as-thermoplastically formed Au surface indicates the grain size of the bulk Au is on the order of 10^1 μm.

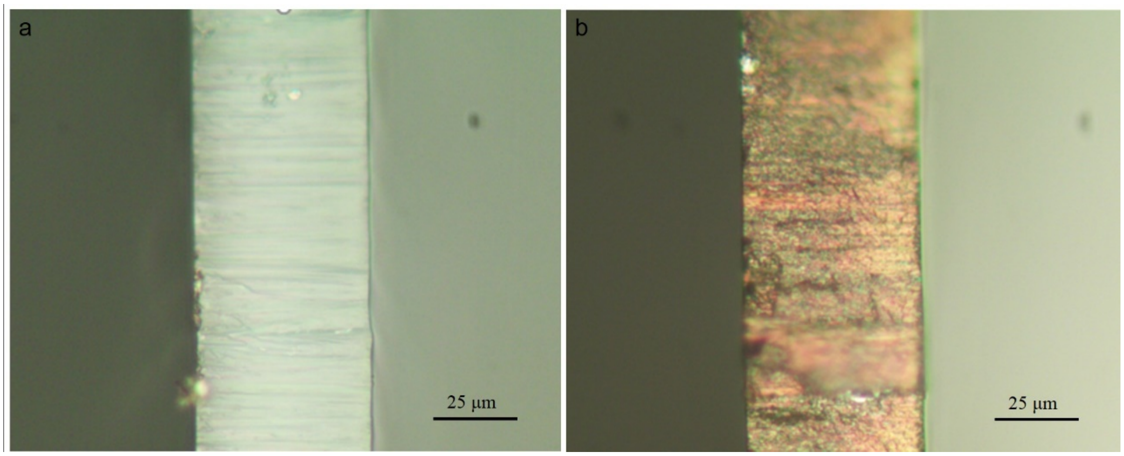

Supplementary Figure 5| Replicated 25 nm Ag nanowire arrays with extremely high aspect ratios. a-b, Optical micrographs shows cross-section of an Al_2O_3 template before (**a**) and after (**b**) superplastic nanoimprinting with Ag.

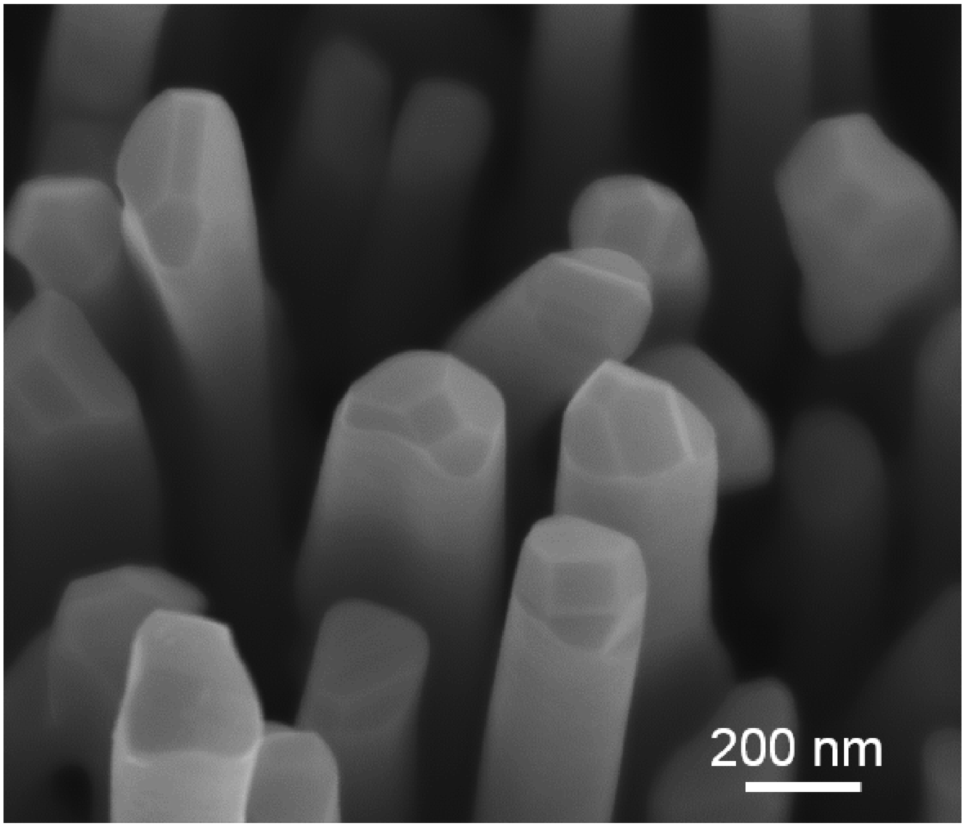

Supplementary Figure 6| SEM image of imprinted Au nanorods with diameters of ~ 200 nm. Where the sample stage was tilted for 15 degrees. The clear crystal facets and regular shapes observed at the top of Au nanorods show their excellent crystallinity.

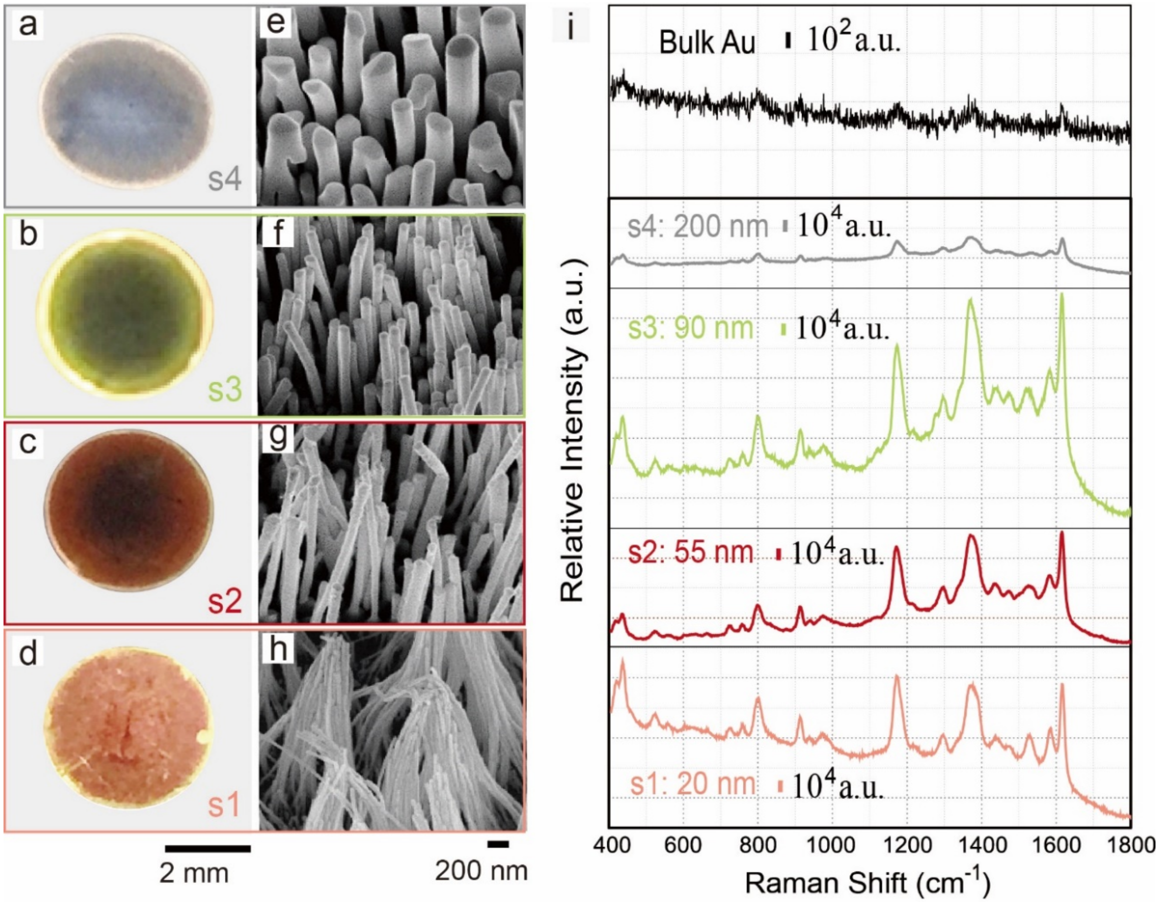

Supplementary Figure 7| Surface-enhanced Raman scattering spectra. a-d, Optical imaging as-thermoplastically formed Au/Al_2O_3 template combinations, where the sizes of replicated Au nanowires are 20 nm (s1), 55 nm (s2), 90 nm (s3) and 200 nm (s4), respectively. The corresponding Au nanowire arrays at the center of samples s1-s4 are characterized by SEM (**e-h**). **i**, SERS spectra of 1.0×10^{-5} M crystal violet adsorbed on the surfaces of samples s1-s4 and a reference bulk Au (see Methods section). Comparing with the reference bulk Au sample, all of the samples (s1-s4) with replicated Au nanowire arrays show drastically enhanced Raman signatures.

Supplementary References

1. Persaud, I. & Grossman, W. E. Surface - enhanced Raman scattering of triphenylmethane dyes on colloidal silver. *J Raman Spectrosc* **24**, 107-112 (1993).
